# Supplementary material for: Study on the Preparation, Characterization, and Stability of Freeze-Dried Curcumin-Loaded Cochleates
Source: Foods. 2022 Feb 28;11(5):710. doi: 10.3390/foods11050710 (PMC8908975; doi:10.3390/foods11050710)
Supplement: Supplementary file 1 [file foods-11-00710-s001.zip › foods-1583136-supplementary.pdf]

The confirmation of Cochleates: When 80 μL $CaCl_2$ (50 mM) was dropped, we could find many CUR-Cochs on the glass slide, and the topography of it was long cylindrical tube.

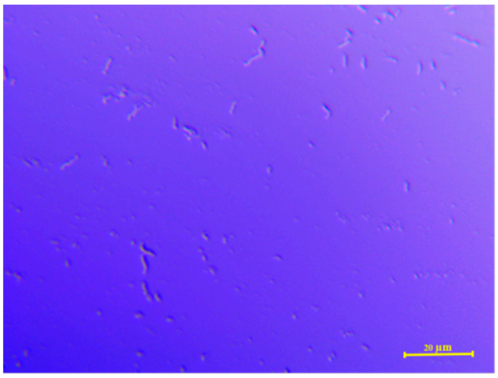

Figure S1 The morphology of CUR-Cochs through an optical microscope observation (80 μL 50 mM $CaCl_2$ solution)

HPLC: The chromatogram of the reference solution was shown in Figure 2, and the retention time of CUR was 6.7 minutes.

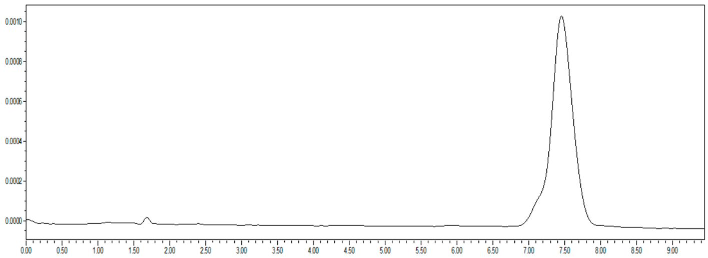

Figure S2 HPLC analysis of CUR solution.

Freeze-drying protective agents Optimizing: Study on the freeze-drying process of CUR-Cochs. We could find that the mannitol group had the best effect which the sample powder did not shrink in volume and was fine. However, the volume of the samples in the lactose and trehalose groups shrank to a certain extent after freeze-drying. The most severe contractions are the glucose group.

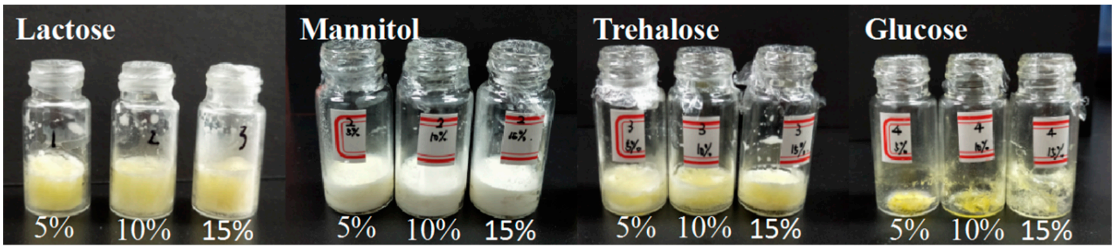

Figure S3 Freeze-dried CUR-Cochs containing different protective agents (5%, 10% and 15% (w/v) respectively).
